# Professor C. R. Agnew

**Published:** 1888-05

**Authors:** 


### OBITUARY.

The Death of Professor Cornelius Rea Agnew.—In the death of Professor Agnew, not alone New York, but the whole medical profession, has sustained a great loss. He was esteemed at home and abroad as a representative medical man. A contemporary truly says of him, he was "beloved by all who knew him, esteemed wherever his name was known, uniting, as few physicians have done, so much learning with so many noble and generous traits of character, so much modesty with such self-sacrifice in the hour of duty, and has left vacant the place in the profession that he has so long honored. Whether in his home, among his patients, in the lecture room, before his colleagues, or in the service of his country, he was the same calm, dignified, learned, charming, Christian gentleman."

